# Magnetic resonance imaging assessment of the therapeutic effect of combined electroacupuncture and stem cells in acute peripheral nerve injury

**DOI:** 10.3389/fncel.2022.1065557

**Published:** 2022-12-20

**Authors:** Yueyao Chen, Zhongxian Pan, Fanqi Meng, Xuewen Yu, Qian Xu, Leyu Huang, Qiumei Liang, Yanglei Wu, Xiaofeng Lin

**Affiliations:** ^1^Department of Radiology, Shenzhen Traditional Chinese Medicine Hospital (The Fourth Clinical Medical College of Guangzhou University of Chinese Medicine), Shenzhen, China; ^2^Department of Pathology, Shenzhen Traditional Chinese Medicine Hospital (The Fourth Clinical Medical College of Guangzhou University of Chinese Medicine), Shenzhen, China; ^3^Siemens Healthineers, Beijing, China; ^4^Department of Nuclear Medicine, The Seventh Affiliated Hospital, Sun Yat-sen University, Shenzhen, China

**Keywords:** magnetic resonance imaging, diffusion tensor imaging, electroacupuncture, mesenchymal stem cells, netrin-1, peripheral nerve injury

## Abstract

**Objectives:** This study aimed to evaluate the therapeutic effect of a combination of Bone Mesenchymal stem cells (BMSCs) transplantation and Electroacupuncture (EA) for acute sciatic nerve injury in rats using magnetic resonance.

**Methods:** Ninety-two male adult healthy Sprague-Dawley rats were randomly divided into the EA+BMSCs group, EA group, MSCs group, and PBS group (control). Electroacupuncture was performed on a rat receiving EA treatment at Huantiao (GB30) and Zusanli (ST36). T2 values and diffusion tensor imaging (DTI) derived from multiparametric magnetic resonance imaging (MRI), histological assessments, and immunohistochemistry was used to monitor nerve regeneration. Walking track analysis was used to assess nerve functional recovery. Repeated-measures one-way analysis of variance was used to evaluate the significance of T2, DTI, and SFI values among the four groups. One-way analysis of variance was used for comparing the histological characteristics. Bonferroni test was used for multiple pairwise comparisons at each time point.

**Results:** In terms of FA, the EA+BMSCs and EA groups had faster recovery than PBS (control) in all time points after surgery, and the EA+BMSCs group recovered better than the BMSCs group at 3 weeks (*P* ≤ 0.008). FA values were higher in the EA group than in the BMSCs group at 4 weeks (*P* ≤ 0.008). In terms of RD, the EA+BMSCs group recovered better than the BMSCs group at 2 and 4 weeks (*P* ≤ 0.008). Immunofluorescence staining for axon guidance molecule netrin-1 revealed that it was significantly higher in the EA+BMSCs subgroup and EA subgroup than it was in the control (PBS) subgroup at 1–3 weeks (*P* < 0.001). Immunofluorescence staining for S100 showed the continuity of nerve fibers recovered more quickly in the EA+BMSCs subgroup than in the BMSCs subgroup.

**Conclusion:** Our research revealed that a combination of MSCs and EA can provide both topological and biomolecular guidance to promote axonal extension, myelin regeneration, and functional recovery after PNI. EA not only promotes nerve repair on its own, but also enhanced the beneficial effects of stem cell treatment and the secretion of netrin 1, a guidance regeneration factor, and promotes the orderly growth of nerve fibers. These PNI repairs could be monitored non-invasively and *in situ* by MRI. The FA and RD values derived from MRI could be sensitive biomarkers to reflect the PNI repair process.

## Introduction

Peripheral nerve injury (PNI) is a clinical condition characterized by sensory and motor deficits. Bone mesenchymal stem cell (BMSC) transplantation has emerged as a promising candidate strategy for the treatment of PNI due to their multipotency, regeneration promotion, secretion of paracrine factors, immunomodulatory properties, and ease of isolation and expansion (Dun and Parkinson, 2017; Yu et al., 2019). Many animal studies and preliminary clinical trials have investigated the efficacy of BMSCs in PNI therapy. Recent clinical trials have indicated that the therapeutic benefit of BMSCs for treating PNI patients remains limited, despite the success of BMSCs in improving nerve injury outcomes in animals (Mathot et al., 2019). In addition, due to abnormal stimulation of axonal buds, BMSC transplantation may result in atypical, non-linear nerve growth patterns (Sullivan et al., 2016).

Electroacupuncture (EA) combines conventional acupuncture with low-voltage electricity. It has been widely utilized for PNI rehabilitation due to its relative simplicity, affordability, and safety compared to conventional therapies (Wu et al., 2021). In addition, increasing evidence has shown that EA can play a beneficial therapeutic role in peripheral neuropathy by rapidly improving nerve function, reducing edema, down-regulating inflammation, and exerting analgesic effects (Ahn et al., 2019).

Notably, studies on cerebral ischemic disease have revealed that grafted BMSCs and EA treatment significantly enhanced the functional recovery of motor and cognitive deficits compared to BMSCs or EA treatment alone. Furthermore, treatment with EA could promote the differentiation of BMSCs into neuron-like cells and migration to injured areas following tissue damage, and enhance growth factor and cytokine expression, as well as cell survival (Emelyanov et al., 2016; Salazar et al., 2017; Ahn et al., 2019). Therefore, EA plus electromagnetic therapy could effectively repair neural and nerve injuries.

Netrin-1 was the first axon guidance molecule discovered in vertebrates and has a strong chemotropic role in axon guidance, cell migration, morphogenesis, and angiogenesis (Dun and Parkinson, 2017). Recent research has shown that the netrin-1 mRNA and protein are expressed in the adult rat sciatic nerve and that Schwann cells are the predominant cell type that secretes netrin-1 (Madison et al., 2000; Dun and Parkinson, 2020). In addition, it is upregulated in distal nerve segment Schwann cells after peripheral nerve injury. Thus, netrin-1 plays a positive role in promoting peripheral nerve regeneration. Whether BMSCs and EA intervention can promote the secretion of netrin-1, thus promoting nerve regeneration and improving function, is an innovation proposed in this study.

Monitoring the fate of therapeutic cells and modalities is typically performed through postmortem histological analysis at predetermined time points, which is laborious and invasive and cannot reflect the longitudinal changes or effect of nerve repair in the same living organism. MRI is a powerful non-invasive diagnostic tool for evaluating PNI and nerve tissue regeneration through direct visualization of peripheral nerve changes (Zheng et al., 2021). Quantitative MRI parameters, such as T2 values and diffusion tensor imaging (DTI) metrics, can detect axonal and myelin information during recovery at multiple time points (Chen et al., 2017; Martín et al., 2017; Zheng et al., 2018). In this study, rat sciatic nerve repair was evaluated using multiparametric MRI, T2 mapping, and DTI. It was hypothesized that combining BMSCs and EA could provide topological and biomolecular guidance to promote axonal extension, myelin regeneration, and functional recovery following PNI. MRI could also be used to monitor PNI repair non-invasively and *in situ*.

## Materials and Methods

### Animals and surgery

All interventions and animal care procedures were performed following the Guangzhou University of Traditional Chinese Medicine’s (Guangzhou, China) Guidelines and Policies for Animal Surgery. They were approved by the Institutional Animal Use and Care Committee. The Animal Core Facility of Jennio Biotech Co., Ltd. (Guangzhou, China) supplied 92 adult Sprague-Dawley rats weighing 220 g each. The rats were housed in a conventional animal facility with a 12-h on/off light cycle and free access to conventional food and water. All rats sustained an acute crush injury to the left sciatic nerve. Each animal’s right sciatic nerve, serving as control, was examined without damaging it.

After administering sodium pentobarbital intraperitoneally at a dose of 30 mg per kilogram of body weight (Sigma-Aldrich; St. Louis, MO), animals were placed in the prone position and the left sciatic nerve was exposed using a blunt splitting technique. A 5-mm-long crush injury was made in the middle of the nerve trunk using hemostatic forceps (Jinzhong Medical Devices Company, Shanghai, China), which were closed to the tightest gear and held in place for 1 min with a holding force of approximately 150 *g* to ensure that all nerve fascicles were completely disrupted with an intact epineurium, as observed under a stereomicroscope (Chen et al., 2017).

After surgery, the animals were randomly divided into four groups: A, EA + BMSCs group; B, EA group; C, BMSCs group; and D, PBS group (control). Each group was separated into two subgroups for functional assessment (A1, B1, C1, and D1; *n* = 8 in each subgroup) and histological analysis (A2, B2, C2, and D2; *n* = 15 in each subgroup). SD rat adolescent bone marrow MSCs were collected and grown as previously described (Chen et al., 2017). On the second day following injury, animals in groups A and B underwent electroacupuncture by stimulating Huantiao (GB30) and Zusanli (ST36; Perreault et al., 2021; Yu et al., 2022). In addition, at the time of modeling, animals in groups A and C received subepidermal microinjections of 5 × 10^5^ BMSCs suspended in phosphate-buffered saline (PBS) with a final volume of 3.0 μl. In contrast, animals in group D received the same volume of PBS as the controls.

**Figure 1:**
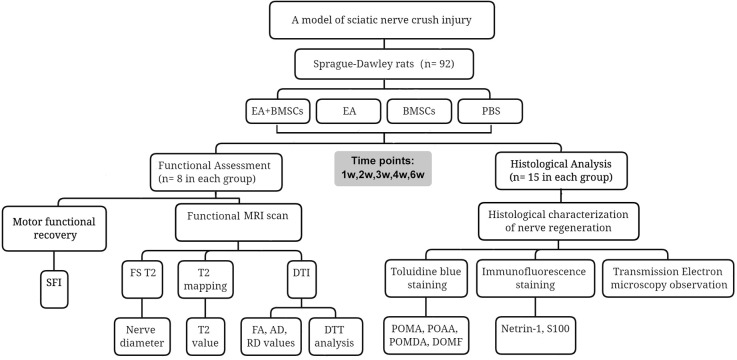
Study timeline and flowchart.

As previously described, microinjections were administered in the epicenter of the lesion using a Hamilton 32-G needle attached to a 10-L syringe (Hamilton Company; Reno, NV). To collect longitudinal MR data, animals in subgroups A1, B1, C1, and D1 underwent serial MR imaging before surgery (0 weeks) and one (cell transplantation), 2, 3, and 6 weeks after surgery. In addition, animals from subgroups A2, B2, C2, and D2 were used to collect histological data; at each time point of 1, 2, 3, 4, and 6 weeks following MR imaging, three from each subgroup were chosen at random and sacrificed for histological analysis. The study design is shown in Figure 1.

### EA treatment

For groups A and B that underwent electroacupuncture treatment, acupuncture needles (0.18 mm and 13 mm) were placed into the acupoints 1 day after the modeling. Groups C and D were minimally restrained in the same manner as the acupuncture group, but needles were not inserted. Connecting a global pulse treatment device (Model G6805-C; Shanghai Huayi Medical Instrument Co., LTD, Shanghai, China) with a frequency of 2/15 Hz, a 20-mA current was used to stimulate acupoints with disperse-dense waves. Mild contraction of the hindlimb muscles was appropriate for this type of stimulus. The positive pole was linked to Huantiao (GB30) on the damaged side of the rats. On the wounded side, Zusanli was connected to the negative pole (ST36). Each of the treatments mentioned above was administered daily for 15 min for 1 week, for six consecutive courses, with 2 days of rest between each course.

### MR imaging

Rats were scanned at 3T after being put to sleep with 7% chloralhydrate (5 ml/kg, intraperitoneal injection; MAGNETOM Prisma, Siemens Healthcare, Erlangen, Germany). Each rat was prone in a rat coil (6-cm diameter, 8-channel, Suzhou Medcoil Healthcare Co., Ltd.), and its limbs were secured with medical adhesive tape to prevent further movement. Both hind limbs were symmetrically positioned.

Parameters of acquisition: Coronal FS T2WI was obtained with repetition time (TR) = 3,000 ms, echo time (TE) = 67 ms, resolution = 100 × 100, field of view (FOV) = 80 mm × 80 mm, Average = 1, slice thickness = 1.0 mm, slices = 20, and voxel size = 0.2 mm × 0.2 mm × 1.0 mm. Coronal T2-mapping was obtained using a multi-slice, multi-echo spin-echo sequence with TR = 1,400 ms, TE = 17–80 ms, resolution = 256 × 256, FOV = 80 mm × 80 mm, Average = 1, slice thickness = 1.0 mm, slices = 10, and voxel size = 0.3 mm × 0.3 mm × 1.0 mm. Axial DTI was obtained using an echo planar imaging (EPI) sequence with the following acquisition parameters: TR = 3,500 ms, TE = 72 ms, slice thickness = 1.5 mm, slices = 20, *b*-value = 0, 800 s/mm^2^, FOV = 70 mm × 70 mm, Average = 3, voxel size = 0.3 mm × 0.3 mm × 1.5 mm; resolution = 100 × 100, EPI factor = 100, flip angle = 90, diffusion mode: MDDW, and diffusion directions = 20. Twenty slices were acquired to cover the damaged sciatic nerve, including the proximal stump, crush lesion, and distal stump. The plane’s center was placed near the epicenter of the lesion. Finally, the total DTI scan time was 4 min and 6 s.

### Imaging analysis

Nerve morphological changes were observed on fat-suppressed T2WI in a blinded manner by two authors in consensus (*** and *** with 10 and 8 years of experience with musculoskeletal MR imaging, respectively). The size of the diameter of the distal stumps of the nerve is measured so that the degree of nerve edema can be recorded. At the same time these two authors independently measured T2 relaxation times of the distal stumps on T2-mapping in a blinded manner using the region of interest (ROI) technique, as previously described (Liao et al., 2012; Chen et al., 2017). In brief, a rectangular ROI with a minimal size of 40 pixels covering a 6-mm proximal segment of the distal stump was placed within the nerve and along the course of the nerve. An effort was made to avoid including fatty tissue, edema, or muscle in the measured volume. The average values from the two datasets were used for analysis.

DTI data were uploaded to the workstation (Syngo Via 2, Siemens) and examined by two authors (*** and ***, each with 10 years of experience with DTI) while isolated and blinded. The diffusion tensor was generated and diagonalized on a pixel-by-pixel basis to provide the eigenvectors and values from which the FA, AD, and RD values were derived for each voxel (Lehmann et al., 2010; Chen et al., 2014). Using ROI data, the DTI metrics of the distal stumps were determined quantitatively. Transverse DTI data were fused with coronal T2-weighted images to confirm that ROIs in the distal stump were positioned correctly and consistently.

A 6-mm-long stump distal to the crushing edge on the three adjacent slices was manually outlined within the ROIs. Three measurements were averaged, and the two mean dataset values were used for analysis. The ROIs were positioned as precisely as possible to minimize the partial volume impact. Tractography was obtained using the fiber tracking analysis software accessible on the same machine. Reconstruction of diffusion tensor tractography (DTT) was performed using a multiple ROI technique (Breitenseher et al., 2015). Briefly, at least two ROIs were manually established at various slice sites along the damaged sciatic nerve on the DTI axial images. One was placed at the proximal stump slice and the other at the distal stump slice. The passage of fibers *via* various ROIs was also seen. FA was set to 0.12, the maximum fiber angle was 50 degrees, and the minimum fiber length was 15 mm (Takagi et al., 2009).

### Motor functional assessment

The motor functional recovery of the sciatic nerve was evaluated blind and in consensus in groups A1, B1, C1, and D1 using walking track analysis performed at each time point prior to MR imaging by two authors (*** and ***, each with 3 years of experience with sciatic nerve functional assessment). As a measure of nerve locomotor dysfunction, the sciatic functional index (SFI) was established (Bain et al., 1989).

### Histology

After MR imaging at specific periods, animals in groups A2, B2, C2, and D2 were euthanized by transcardial perfusion with PBS followed by 4% paraformaldehyde in 0.1 M PBS (pH 7.4). The central and distal stumps of the damaged nerves were removed, fixed in 4% paraformaldehyde for 1 h, and maintained in 20% sucrose solution until examination. Transverse semi-thin sections (2 μm thickness) were prepared from the distal end of the tissue specimens and stained with toluidine blue to detect nerve degeneration (Feirabend et al., 1998). Contiguous 15-μm-thick longitudinal sections were obtained at the proximal end of the tissue specimens and processed for netrin-1, and S100 immunofluorescence staining occurred to assess axonal regeneration, as previously described (Zhang et al., 2014; Salameh et al., 2018). For netrin-1, an objective magnification of ×400 was used to obtain digital images of the entire cross-sectional area of the nerve (1,388 × 1,040 pixels, 3.9 pixels/μm) on a microscope (Olympus BX60; Japan) for detailed histological quantification. Of these images, five randomly selected images (585 × 468 pixels, 3.9 pixels/μm) were analyzed. Image Pro Plus software was used to perform an analysis to determine the integrated optical density (IOD) of netrin-1 (Sun et al., 2017; Huang et al., 2021). The final value used for statistical analysis represented the mean of five measured images per nerve segment and animal.

To quantify toluidine blue staining, sections of the distal stumps were analyzed morphometrically. In brief, an objective magnification of ×1,000 was used to obtain digital images of the entire cross-sectional area of the nerve (1,920 × 1,080 pixels, 13.7 pixels/μm) on a microscope (Olympus BX60; Japan) for detailed histological quantification. Of these images, five randomly selected measured images (1,644 × 959 pixels, 13.7 pixels/μm) were analyzed. ImageJ software was used to determine the percentage of axon area (POAA), percentage of myelin area (POMA), percentage of myelin debris area (POMDA), and diameter of myelinated fiber (DOMF). The final value used for statistical analysis represented the mean of five measuring images per nerve segment and animal.

To observe the microstructural changes of the axon and myelin sheath during the regeneration of the injured rat sciatic nerve in different subgroups, transmission electron microscopy (TEM) was performed (Liu et al., 2019).

### Statistical analysis

Repeated-measures one-way analysis of variance (ANOVA) was used to evaluate the significance of DTI and SFI values among the four groups in the first subgroup of animals who underwent MRI and functional recovery assessments. The DTI and SFI values were compared between these subgroups using a Bonferroni test for multiple pairwise comparisons at each time point (zero to about 6 weeks post-surgery) to determine the effects of EA. Histological characterization was compared between subgroups in the second group of animals, who were subjected to histologic analysis, using one-way analysis of variance followed by a Bonferroni *post-hoc* test for multiple pairwise comparisons. Statistical analyses were performed using SPSS version 22.0. All data were presented as means ± standard deviation. A two-sided *P*-value of 0.05 or less indicated a significant difference, and the significance level was *P* ≤ 0.008 (0.05/6) for *post-hoc* Bonferroni test (six comparisons were made between these four groups).

## Results

### MRI monitoring of PNI repair *in vivo*

#### Nerve diameter and T2 values

**Figure 2:**
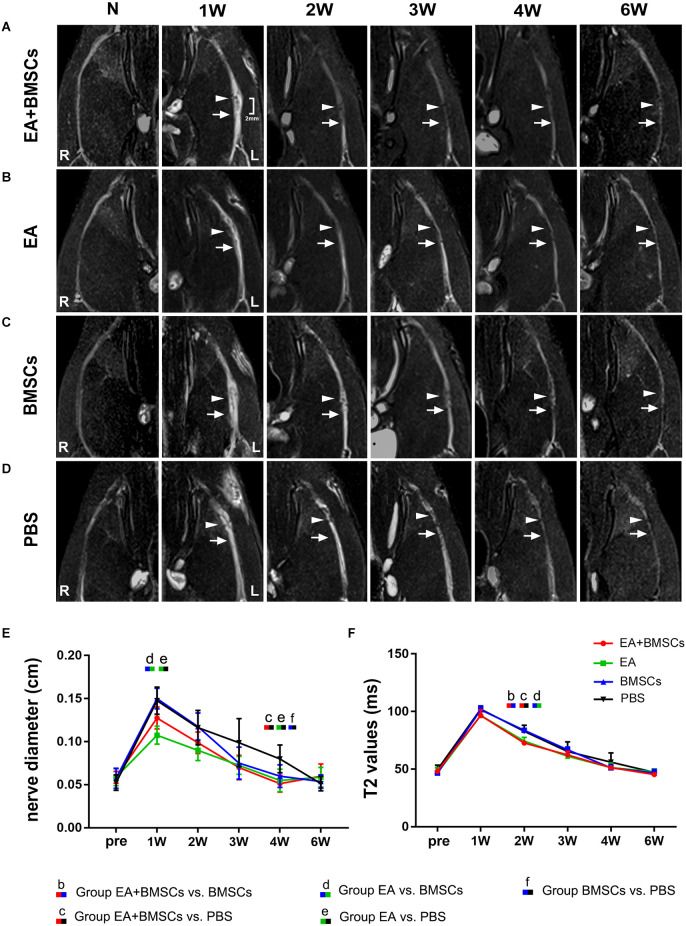
Sequential FS-T2WI of injured and uninjured contralateral sciatic nerves in rats. **(A–D)** MRI images depicting the injured nerves on the left side of the EA + BMSCs, EA, BMSCs, and PBS subgroups and the uninjured nerves (control group) on the opposite side. Injured nerves in all subgroups exhibited enlarged and hyperintense signals in the proximal and distal portions, which decreased gradually. BMSCs were injected into the crushed section (indicated by arrowheads). R: right, L: left, W: week. **(E)** Nerve diameter in the distal stumps of the injured nerves of four subgroups up to 6 weeks post-surgery. **(F)** T2 values in the distal stumps of the injured nerves of four subgroups up to 6 weeks post-surgery. The nerve diameter and T2 values of acquisition points within groups were compared by using repeated-measures one-way ANOVA with Bonferroni *post-hoc* testing, and statistically significant differences are listed in Tables s 1, 2 respectively.

After surgery, the injured sciatic nerves in the EA + BMSCs, EA, BMSCs, and PBS subgroups were visualized by FS-T2WI (Figures 2A–D). The contralateral side (right side) shows T2 images of normal sciatic nerves. In the 1st week after surgery, the edema of the damaged and distal stumps of the damaged nerves was severe, displaying an enlarged nerve diameter and hyperintense T2 signals in all groups. Subsequently, as the edema gradually subsided, the nerve diameter decreased. For repeated-measures ANOVA, there was a significant interaction between time, treatment, and time*group for nerve diameter (all *P* < 0.001) and T2 values (*P* < 0.001 for time, *P* = 0.003 for treatment, and *P* < 0.001 for time*treatment). At 3 and 4 weeks, the groups treated with EA and EA + BMSCs showed a more rapid reduction in swelling. At 1 week, the nerve diameter of the EA subgroup was less than that of the BMSCs (*P* ≤ 0.008) and PBS groups (*P* ≤ 0.008). At 4 weeks, the nerve diameter of the EA + BMSCs group was significantly smaller than that of the PBS grouping (*P* ≤ 0.008). Six weeks later, the morphology of all nerves had been restored. In addition, a smaller nerve diameter was detected in the PBS group compared to the contralateral side due to nerve atrophy after chronic injury (Figure 2E, Table 1).

**Table 1:** Nerve diameter in EA + BMSCs, EA, BMSCs, and PBS [M (P25, P75)].

**MRI**	**Time points (weeks)**	**EA + BMSCs**	**EA**	**BMSCs**	**PBS**	***P* ≤ 0.008**
Nerve diameter	Pre	0.06 (0.05, 0.06)	0.06 (0.05, 0.06)	0.06 (0.05, 0.06)	0.06 (0.04, 0.06)	-
	1	0.13 (0.12, 0.14)	0.11 (0.10, 0.11)	0.15 (0.14, 0.16)	0.14 (0.14, 0.16)	de
	2	0.10 (0.09, 0.11)	0.09 (0.08, 0.10)	0.12 (0.11, 0.13)	0.12 (0.10, 0.14)	-
	3	0.07 (0.06, 0.08)	0.08 (0.06, 0.08)	0.07 (0.06, 0.09)	0.09 (0.08, 0.13)	-
	4	0.06 (0.04, 0.06)	0.06 (0.04, 0.06)	0.06 (0.05, 0.06)	0.08 (0.07, 0.09)	cef
	6	0.06 (0.05, 0.06)	0.06 (0.05, 0.06)	0.06 (0.05, 0.06)	0.05 (0.04, 0.06)	-

MRI, magnetic resonance imaging; EA, electroacupuncture; BMSCs, bone mesenchymal stem cells; PBS, phosphate-buffered saline; *P* ≤ 0.008, *post-hoc* analysis with Bonferroni correction. Statistically significant difference between: ^a^Group EA + BMSCs vs. EA. ^b^Group EA + BMSCs vs. BMSCs. ^c^Group EA + BMSCs vs. PBS. ^d^Group EA vs. BMSCs. ^e^Group EA vs. PBS. ^f^Group BMSCs vs. PBS.

**Table 2:** MRI values in EA + BMSCs, EA, BMSCs, and PBS (mean ± SD).

**MRI**	**Time points (weeks)**	**EA + BMSCs**	**EA**	**BMSCs**	**PBS**	***P* ≤ 0.008**
T2	Pre	48.21 ± 2.0	46.84 ± 4.32	46.51 ± 1.75	49.65 ± 3.77	-
	1	96.55 ± 4.91	96.50 ± 6.04	101.43 ± 3.08	102.04 ± 2.73	-
	2	72.31 ± 1.52	74.33 ± 3.22	83.73 ± 1.43	82.81 ± 5.31	bcd
	3	62.30 ± 3.19	61.21 ± 5.53	66.78 ± 2.36	65.32 ± 8.36	-
	4	51.75 ± 1.38	51.26 ± 3.89	51.11 ± 2.91	56.05 ± 8.01	-
	6	45.83 ± 1.20	47.68 ± 2.41	45.92 ± 2.38	47.19 ± 2.90	-
						
FA	Pre	0.637 ± 0.005	0.625 ± 0.014	0.633 ± 0.004	0.627 ± 0.014	-
	1	0.409 ± 0.016	0.405 ± 0.012	0.406 ± 0.016	0.397 ± 0.013	-
	2	0.454 ± 0.018	0.447 ± 0.016	0.433 ± 0.014	0.412 ± 0.015	ce
	3	0.508 ± 0.025	0.492 ± 0.023	0.472 ± 0.018	0.455 ± 0.019	bce
	4	0.530 ± 0.006	0.525 ± 0.028	0.499 ± 0.006	0.492 ± 0.014	bcde
	6	0.644 ± 0.005	0.618 ± 0.009	0.614 ± 0.004	0.599 ± 0.013	abcef
						
RD	Pre	0.643 ± 0.009	0.646 ± 0.054	0.655 ± 0.024	0.653 ± 0.031	-
	1	0.867 ± 0.020	0.880 ± 0.021	0.880 ± 0.021	0.889 ± 0.018	-
	2	0.764 ± 0.022	0.797 ± 0.027	0.810 ± 0.029	0.843 ± 0.029	bef
	3	0.726 ± 0.021	0.745 ± 0.032	0.747 ± 0.024	0.788 ± 0.031	cef
	4	0.711 ± 0.011	0.722 ± 0.014	0.731 ± 0.017	0.753 ± 0.013	bcef
	6	0.639 ± 0.015	0.645 ± 0.020	0.648 ± 0.015	0.658 ± 0.016	-
						
AD	Pre	2.088 ± 0.073	2.071 ± 0.039	2.080 ± 0.043	2.072 ± 0.049	-
	1	1.835 ± 0.072	1.822 ± 0.062	1.825 ± 0.062	1.819 ± 0.041	-
	2	1.886 ± 0.080	1.811 ± 0.041	1.859 ± 0.039	1.826 ± 0.053	-
	3	1.924 ± 0.073	1.867 ± 0.035	1.904 ± 0.044	1.829 ± 0.061	c
	4	1.946 ± 0.030	1.903 ± 0.090	1.920 ± 0.059	1.870 ± 0.043	c
	6	2.053 ± 0.049	1.966 ± 0.070	2.025 ± 0.064	1.894 ± 0.083	cf

EA, electroacupuncture; BMSCs, bone mesenchymal stem cells; SD, standard deviation; *P* ≤ 0.008, *post-hoc* analysis with Bonferroni correction; FA, fractional anisotropy; RD, radial diffusivity; AD, axial diffusion; PBS, phosphate-buffered saline. Statistically significant difference between: ^a^Group EA + BMSCs vs. EA. ^b^Group EA + BMSCs vs. BMSCs. ^c^Group EA + BMSCs vs. PBS. ^d^Group EA vs. BMSCs. ^e^Group EA vs. PBS. ^f^Group BMSCs vs. PBS.

The T2 signals of all subgroups reverted to normal (comparable to the contralateral side) 6 weeks after surgery. However, the temporal dependency of T2 values in the distal stumps of the wounded nerves differed across the EA + BMSCs, EA, BMSCs, and PBS subgroups (Figure 2F). After 1 week, the T2 readings of all groups climbed fast and peaked at similar levels. Then, they declined gradually but at varying rates (slopes are given in Figure 2F). At 2 weeks, both groups treated with electroacupuncture recovered quicker than other subgroups (both *P* ≤ 0.008). In addition, at 2 weeks, the T2 levels of the EA subgroup were lower than those of the BMSCs subgroup (*P* ≤ 0.008; Table 2).

#### DTI metrics of nerves

**Figure 3:**
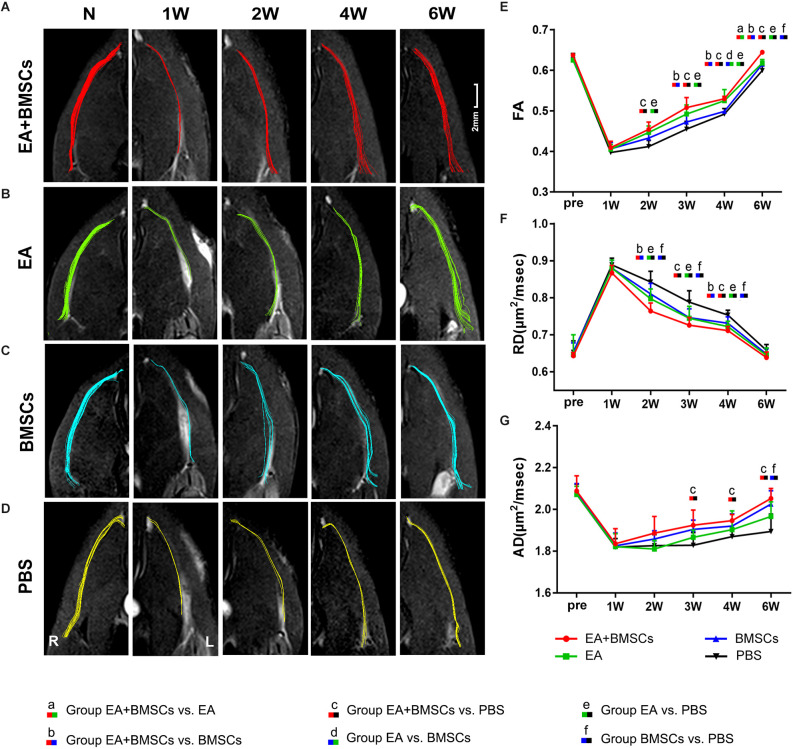
DTI of injured sciatic nerves in rats. **(A–D)** DTT of injured sciatic nerves compared to uninjured contralateral nerves after surgery. Images depicting the injured nerves in the EA + BMSCs, EA, BMSCs, and PBS subgroups on the left and the uninjured nerves (control group) on the right. R: right, L: left, W: week. **(E–G)** Six weeks after surgery, FA, RD, and AD values in the distal stumps of all injured nerves were compared to their respective control values. The FA, RD, and AD values of acquisition points within groups were compared by using repeated-measures one-way ANOVA with Bonferroni *post hoc* testing, and Table 2 lists statistically significant differences. DTI, diffusion tensor imaging; DTT, diffusion tensor tractography; EA, electroacupuncture; BMSCs, bone mesenchymal stem cells; PBS, phosphate-buffered saline; FA, fractional anisotropy; RD, radial diffusivity; AD, axial diffusion.

For repeated-measures ANOVA, there was a significant interaction between time, treatment, and time*group for FA (all *P* < 0.001) and RD (all *P* < 0.001). For AD, there was a significant interaction between time (*P* < 0.001) and treatment (*P* < 0.001), but not between time*group (*P* = 0.077). Two to 6 weeks following surgery, the FA levels rose (Figure 3E), indicating axonal regeneration in the damaged nerves. The FA values of the injured nerves gradually normalized. Both subgroups treated with EA exhibited larger FA values than the EA-untreated group (*P* ≤ 0.008) in the 2nd week after surgery. Among all the groups, the EA + BMSCs group maintained the most significant FA values at 3, 4, and 6 weeks post-operation, followed by the EA group, but the FA values of the PBS group were the lowest. Among them, the EA + BMSCs and EA groups had faster recovery than the PBS (control) group at all time points after surgery in terms of FA. The EA + BMSCs group recovered better than the BMSCs group at 3 weeks (*P* ≤ 0.008). FA values were higher in the EA group than in the BMSCs group at 4 weeks (*P* ≤ 0.008). The FA values of the EA + BMSCs group were higher than the other groups at 6 weeks (*P* ≤ 0.008).

Furthermore, the EA and BMSCs groups had a lower RD value than the PBS (control) group (both *P* ≤ 0.008) approximately 2–4 weeks after surgery. The EA + BMSCs group recovered better than the BMSCs group (*P* ≤ 0.008) at 2 and 4 weeks. In addition, the RD values were lower in the EA + BMSCs group than in the PBS (control) group (*P* ≤ 0.008) at 3 and 4 weeks. AD values were higher in the EA + BMSCs group than in the PBS group (*P* ≤ 0.008) at 3 and 4 weeks, and the AD values of the BMSCs group were higher than in PBS (control) groups (*P* ≤ 0.008) at 6 weeks (Table 2).

Comparing the preoperative measurements, the FA of the EA + BMSCs group value recovered and was higher (*P* = 0.028) at the 6th week, and the AD value recovered to the preoperative level (*P* = 0.332). In the EA group, the FA value recovered to the preoperative level (*P* = 0.326), and the AD value was close to the preoperative level, but there were still differences (0.645 ± 0.020 vs. 0.646 ± 0.054, *P* = 0.017). In the BMSCs group, the FA value had not recovered to the preoperative level (*P* < 0.001), and the AD value recovered to the preoperative level (*P* = 0.126). In the PBS group, neither FA nor AD recovered to the preoperative level (both *P* < 0.05).

#### DTT of nerves

Compared with the uninjured contralateral nerves, the number of projected injured nerve fibers decreased 2 weeks after surgery, as characterized by DTT analysis (Figure 3). The EA + BMSCs and EA groups showed more nerve fibers and more significant trajectories than those implanted without EA treatment at 2–6 weeks after surgery.

### Histological characterization of nerve regeneration

**Figure 4:**
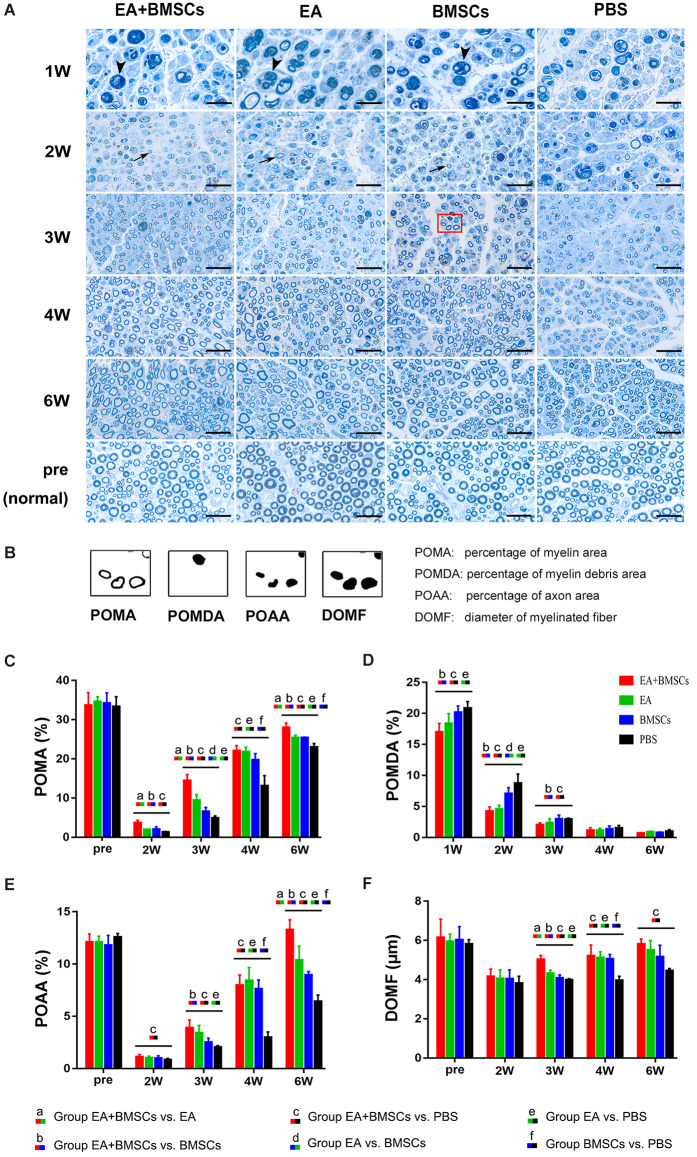
Toluidine blue staining of the distal stumps of injured and uninjured contralateral nerves. **(A)** Representative cross-sectional micrographs exhibiting axonal degeneration and myelin disintegration at 1 and 2 weeks post-surgery. The arrowheads denote the myelin debris, which was removed with nerve repaired. By the second week, new myelin sheaths begin to appear, which were thin and small (arrows). Pronounced axonal regeneration and remyelination were observed at 6 weeks post-operation. W: week. Scale bars = 20 μm. **(B)** Semi-quantitative analysis of the four pathological indicators. **(C–F)** Percentage of myelin area (POMA), percentage of myelin debris area (POMDA), percentage of axon area (POAA), and diameter of myelinated nerve fibers (DOMF) in the distal stumps of the injured nerves of all groups compared to their contralateral uninjured nerves (control group) at about 2–6 weeks post-surgery. The POMA, POMDA, POAA, and DOMF values of acquisition points within groups were compared by using one-way ANOVA with Bonferroni *post-hoc* testing, and statistically significant differences are listed in Table 3.

**Figure 5:**
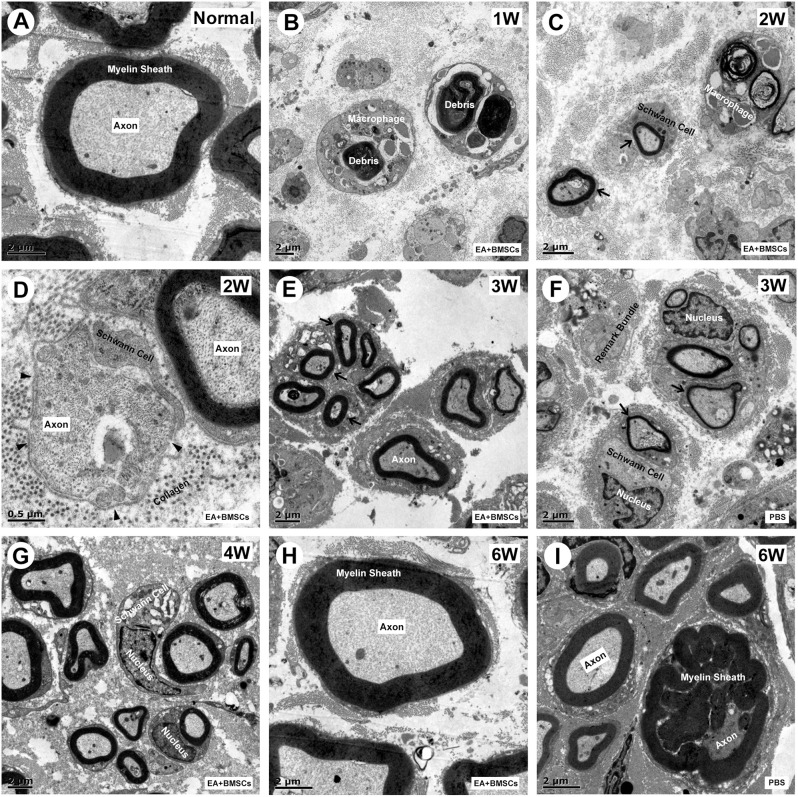
Transmission electron microscopy (TEM) images of cross-sections of the sciatic nerve. **(A)** Cross-section of a myelinated axon of a normal adult mouse sciatic nerve. The central axon (Ax) is surrounded by the myelin sheath (MS), with a thickness of 1.2 to 2 μm. **(B)** At 1-week post-operation, the myelin sheath became creased, strangled, and fell off into fragments. Massive myelin debris was phagocytosed and cleared by the macrophages. **(C,D)** Most debris was cleared at 2 weeks post-operation. The Schwann cells thrived, and their synapses wrapped around the axons to form new myelin sheaths, which were thin in myelin sheaths (< 0.5 μm; black arrow) and small in diameter. In addition, some unmyelinated fibers (black arrowhead) could be seen, with surrounding collagen tissue serving as a scaffold for the extension and winding of Schwann cells during myelin formation **(D)**. **(E,G)** During the period from 3 to 4 weeks after surgery, the number of myelinated nerve fibers, myelin thickness, and the degree of maturity of regenerating nerve fibers in EA + BMSCs group were superior to that in the PBS group **(F)**. **(H,I)** At 6 weeks post-operation, nerve fibers of EA + BMSCs group **(H)** recovered with regenerated axons and myelin sheaths comparable to those of the normal nerves **(A)**, as well as superior fiber diameter and myelin thickness to that of the PBS subgroup **(I)**. Some malformed regenerated fibers were observed in the PBS subgroup, with abnormally thickened myelin sheaths that are convex in the lumen. The axons are compressed and narrow.

One to 2 weeks after surgery, toluidine blue staining of the distal stumps of the damaged nerves indicated extensive myelin loss and axonal degeneration (Figure 4A). All groups demonstrated considerable improvement in myelin debris clearing (Figure 4C), axonal regeneration, and remyelination 2 and 6 weeks after surgery (Figure 4A). Statistically, the EA + BMSCs group displayed the greatest number of regenerated myelinated fibers (Figures 4B,D,E) at 3 and 6 weeks and the greatest myelinated nerve fiber diameter (Figure 4F) at the distal stumps, followed by the EA group (Table 3). TEM revealed ultrastructural modifications after the healing of the sciatic nerve (Figure 5).

**Table 3:** Histological characterizations in EA + BMSCs, EA, BMSCs, and PBS (mean ± SD).

**Histology of the injured nerves**	**Time points (weeks)**	**EA + BMSCs**	**EA**	**BMSCs**	**PBS**	***P* ≤ 0.008**
POMA	Pre	33.68 ± 3.23	34.57 ± 1.27	34.19 ± 2.63	33.31 ± 2.53	-
	2	3.72 ± 0.62	1.97 ± 0.09	2.04 ± 0.61	1.30 ± 0.20	abc
	3	14.46 ± 1.52	9.42 ± 1.45	6.56 ± 1.05	4.89 ± 0.55	abcde
	4	22.05 ± 1.33	21.8 ± 1.17	19.7 ± 1.58	13.08 ± 2.64	cef
	6	27.99 ± 1.15	25.3 ± 0.67	25.3 ± 0.17	22.98 ± 0.94	abcef
						
POAA	Pre	12.09 ± 0.79	12.09 ± 0.58	11.80 ± 0.94	12.59 ± 0.35	-
	2	1.30 ± 0.20	1.00 ± 0.18	0.98 ± 0.24	0.84 ± 0.13	c
	3	3.88 ± 0.78	3.42 ± 0.71	2.50 ± 0.41	2.04 ± 0.16	bce
	4	7.97 ± 0.98	8.42 ± 1.25	7.64 ± 0.85	2.98 ± 0.53	cef
	6	13.27 ± 0.97	10.38 ± 1.32	8.95 ± 0.34	6.41 ± 0.64	abcef
						
POMDA	1	17.00 ± 1.39	20.85 ± 1.59	20.18 ± 1.01	20.85 ± 1.05	bce
	2	4.24 ± 0.69	4.58 ± 0.62	7.08 ± 0.96	8.74 ± 1.48	bcde
	3	2.01 ± 0.33	2.36 ± 0.65	2.98 ± 0.63	2.96 ± 0.18	bc
	4	1.16 ± 0.41	1.16 ± 0.33	1.36 ± 0.49	1.51 ± 0.42	-
	6	0.69 ± 0.09	0.89 ± 0.11	0.75 ± 0.13	1.00 ± 0.23	-
						
DOMF	Pre	6.14 ± 0.94	5.95 ± 0.37	6.02 ± 0.68	5.81 ± 0.23	-
	2	3.98 ± 0.10	4.26 ± 0.45	4.04 ± 0.45	3.81 ± 0.36	-
	3	5.03 ± 0.21	4.32 ± 0.17	4.08 ± 0.17	3.98 ± 0.07	abce
	4	5.21 ± 0.55	5.10 ± 0.31	5.05 ± 0.23	3.96 ± 0.21	cef
	6	5.81 ± 0.26	5.51 ± 0.49	5.16 ± 0.55	4.46 ± 0.11	c

EA, electroacupuncture; BMSCs, bone mesenchymal stem cells; PBS, phosphate-buffered saline; SD, standard deviation; *P* ≤ 0.008, *post-hoc* analysis with Bonferroni correction; POMA, percentage of myelin area; POAA, percentage of axon area; POMDA, percentage of myelin debris area; DOMF, diameter of myelinated fiber. Statistically significant difference between: ^a^Group EA + BMSCs vs. EA. ^b^Group EA + BMSCs vs. BMSCs. ^c^Group EA + BMSCs vs. PBS. ^d^Group EA vs. BMSCs. ^e^Group EA vs. PBS. ^f^Group BMSCs vs. PBS.

Comparing the preoperative measurements, the POAA recovered to the preoperative level in the EA + BMSCs subgroup and EA subgroup at the 6th week (all *P* > 0.05). In the BMSCs group, the POAA had not recovered to the preoperative level (*P* = 0.002). In the PBS group, the POAA recovered to the preoperative level (*P* < 0.001).

**Figure 6:**
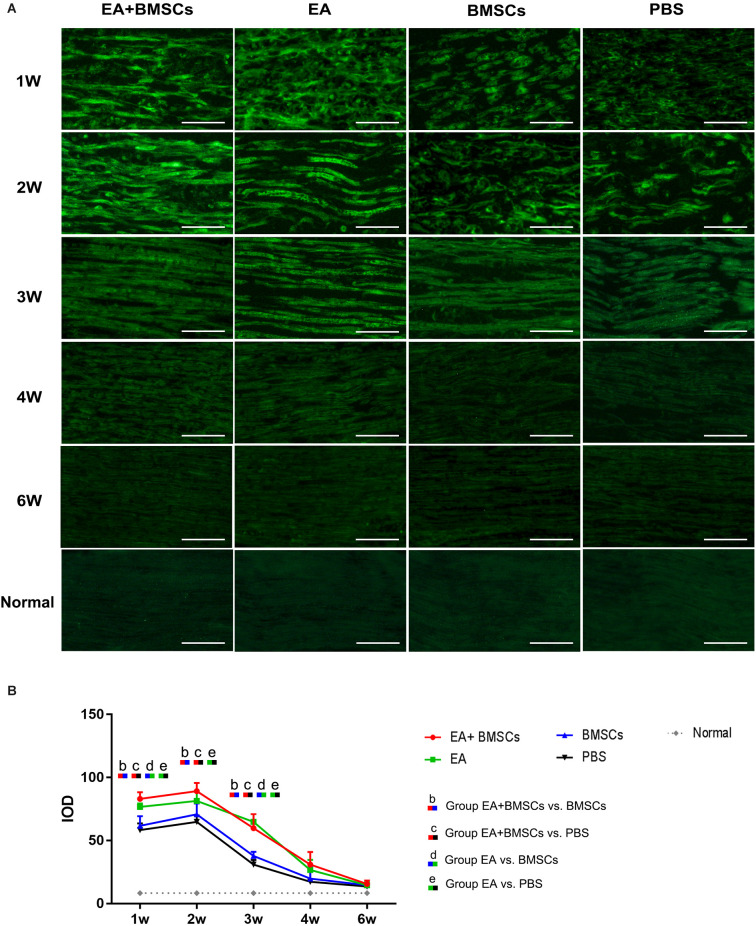
Histology of netrin-1 expression of the injured nerves. Immunofluorescence staining shows that netrin-1 is present in the myelin sheath composed of Schwann cells, therefore when it is secreted, the myelin sheath is colored and outlined **(A)**. The expression of netrin-1 of all subgroups significantly increased in 1–2 weeks. The EA+BMSCs and EA subgroups had higher netrin-1 expression (green) than the BMSCs and control subgroups (PBS treatment) in 1–3 weeks **(B)**. In addition, 4 and 6 week figures had a darker degree of staining with the loss of fiber textures **(A)**, which could be judged as a negative expression of the netrin-1 protein. Scale bar = 50 μm; W: week. The IOD of acquisition points within groups were compared by using one-way ANOVA with Bonferroni *post-hoc* testing, and Table 4 lists statistically significant differences. IOD, integrated optical density.

Immunofluorescence staining for netrin-1 showed that the IOD of netrin-1 in all groups was significantly increased at 1–2 weeks, with a peak at 2 weeks, followed by a rapid decrease at 3 weeks and a gradual decrease to a normal level. As shown in Table 4, the IOD of netrin-1 in the EA + BMSCs and EA subgroups was significantly higher than in the control (PBS) subgroup at 1–3 weeks (*P* < 0.001). In addition, the IOD in the EA + BMSCs subgroup was markedly higher than in the BMSCs subgroup (*P* = 0.000). At 1 and 3 weeks, the netrin-1 expression of the EA subgroup was higher than in the BMSCs subgroup (*P* = 0.001; *P* < 0.001; Figure 6; Table 4).

**Table 4:** IOD values in EA + BMSCs, EA, BMSCs, and PBS (mean ± SD).

**Time points (weeks)**	**EA + BMSCs**	**EA**	**BMSCs**	**PBS**	**Normal**	***P* ≤ 0.008**
1	83.04 ± 5.31	76.72 ± 2.78	61.65 ± 7.62	58.31 ± 5.21	8.33 ± 2.01	bcde
2	89.24 ± 6.60	80.90 ± 6.61	70.82 ± 8.60	64.77 ± 5.11	8.33 ± 2.01	bce
3	59.81 ± 11.26	64.75 ± 6.28	37.86 ± 3.34	30.88 ± 3.63	8.33 ± 2.01	bcde
4	30.94 ± 9.96	26.63 ± 8.07	19.86 ± 8.96	17.34 ± 7.84	8.33 ± 2.01	-
6	15.61 ± 2.85	14.60 ± 2.29	14.56 ± 1.88	13.56 ± 2.24	8.33 ± 2.01	-

IOD, integrated optical density; EA, electroacupuncture; BMSCs, bone mesenchymal stem cells; SD, standard deviation; *P* ≤ 0.008, *post-hoc* analysis with Bonferroni correction. Statistically significant difference between: ^a^Group EA + BMSCs vs. EA. ^b^Group EA + BMSCs vs. BMSCs. ^c^Group EA + BMSCs vs. PBS. ^d^Group EA vs. BMSCs. ^e^Group EA vs. PBS. ^f^Group BMSCs vs. PBS.

**Figure 7:**
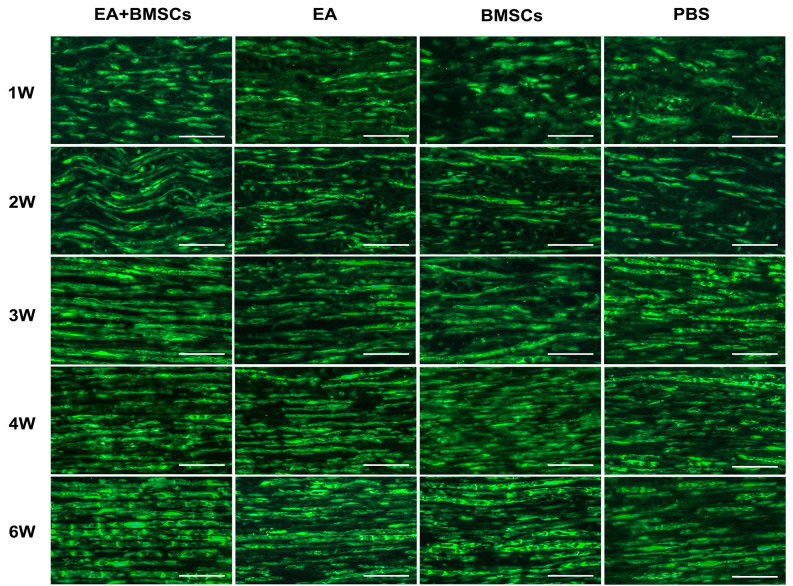
S100 staining of the distal stumps of injured nerves. Immunohistochemical staining of S100 observed under the fluorescence microscope (400×) showed the continuity of the restored, damaged sciatic nerve. In terms of neural regeneration connections, compared with the PBS subgroup, the EA + BMSCs subgroup recovered best. In addition, the EA subgroup was better than the BMSC subgroup. Scale bar = 50 μm; W: week.

Immunofluorescence staining for S100 showed that the continuity of nerve fibers of the injured nerves decreased 1 week post-operation, followed by a gradual recovery in all four subgroups. At 3 weeks post-surgery, the continuity of the injured nerve fibers improved. At 6 weeks post-operation, the continuity of the injured site nerve fibers in the EA + BMSCs and EA subgroups showed almost a full recovery, but not in the BMSCs and control (PBS) subgroups. In addition, the continuity of nerve fibers recovered more quickly in the EA + BMSCs than in the BMSCs subgroup. Finally, the control subgroup (PBS) showed the slowest recovery of the continuity of nerve fibers (Figure 7).

### Motor functional recovery

**Figure 8:**
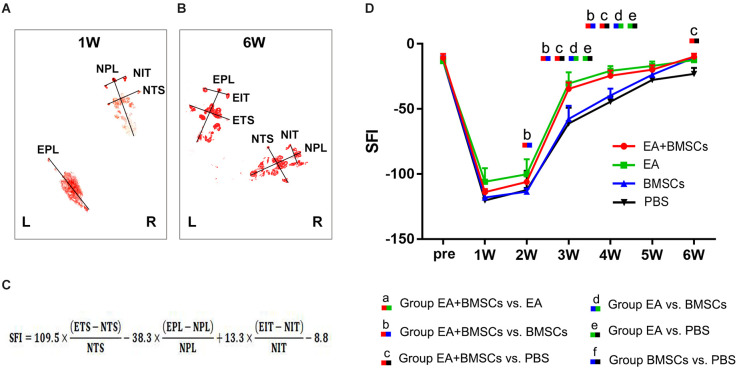
Walking track analysis of the sciatic nerve-injured rats. **(A,B)** Hind paw footprints of the EA + BMSCs subgroup at 1 and 6 weeks post-surgery. **(C)** The formula to calculate the sciatic functional index (SFI) is shown as derived by multiple linear regression (EPL, experimental print length; NPL, normal print length; ETS, experimental toe spread; NTS, normal toe spread; EIT, experimental intermediary toe spread; and NIT, normal intermediary toe spread). **(D)** Sciatic nerve function index (SFI) of the injured side compared with the uninjured contralateral side post-surgery in the four sub-groups. The SFI of acquisition points within groups were compared by using one-way ANOVA with Bonferroni *post-hoc* testing, and statistically significant differences are listed in Table 5.

The recovery of motor function in injured rats was examined using walling track analysis and SFI. The footprints of the wounded rats’ rear paws were documented (Figures 8A–C). For repeated-measures ANOVA, there was a significant interaction between time (*P* < 0.001), group (*P* < 0.001), and time*group (*P* < 0.001) for SFI. According to the SFI formula (Figure 8C), the normal sciatic nerve function index tends to be zero before the injury. The injured animals exhibited a rapid decrease in SFI within the 1st week following surgery, which recovered progressively (Figure 8D). At 2–5 weeks following surgery, the EA + BMSCs group had the highest SFI value, followed by the EA group alone. The SFI values of the EA group were −30.38 ± 8.72 and −20.75 ± 3.66 at 3 and 4 weeks, respectively (Table 5), whereas those of the EA + BMSCs group were −34.57 ± 4.92 and −24.50 ± 4.08, respectively. The respective SFI values of these two groups were higher than those of the BMSCs (−57.69 ± 10.11 and −39.72 ± 5.29) and PBS groups (−61.13 ± 12.23 and −44.47 ± 4.21). In addition, the SFI values of the EA + BMSCs group were higher than those of the BMSCs group at 2 weeks and the PBS group at 6 weeks. These results indicated that the injured nerve with EA or EA + BMSCs treatment provided a more rapid functional restoration at 2–6 weeks post-surgery.

**Table 5:** SFI values in EA + BMSCs, EA, BMSCs, and PBS (mean ± SD).

**MRI**	**Time points (weeks)**	**EA + BMSCs**	**EA**	**BMSCs**	**PBS**	***P* ≤ 0.008**
SFI	Pre	−10.70 ± 3.07	−13.17 ± 3.16	−12.59 ± 3.15	−13.12 ± 3.00	-
	1	−113.97 ± 2.06	−105.9 ± 10.43	−118.10 ± 5.91	−120.33 ± 5.95	-
	2	−106.08 ± 1.35	−100.20 ± 11.55	−113.52 ± 2.42	−112.33 ± 14.80	b
	3	−34.57 ± 4.92	−30.38 ± 8.72	57.69 ± 10.11	−61.13 ± 12.23	bcde
	4	−24.50 ± 4.08	−20.75 ± 3.66	−39.72 ± 5.29	−44.47 ± 4.21	bcde
	5	−19.85 ± 3.39	−16.88 ± 3.47	−23.66 ± 3.67	−27.68 ± 8.26	-
	6	−9.79 ± 2.32	−12.41 ± 2.68	−10.62 ± 1.33	−23.06 ± 4.62	c

SFI, sciatic functional index; EA, electroacupuncture; BMSCs, bone mesenchymal stem cells; SD, standard deviation; MRI, magnetic resonance imaging. *P* ≤ 0.008, *post-hoc* analysis with Bonferroni correction. Statistically significant difference between: ^a^Group EA + BMSCs vs. EA. ^b^Group EA + BMSCs vs. BMSCs. ^c^Group EA + BMSCs vs. PBS. ^d^Group EA vs. BMSCs. ^e^Group EA vs. PBS. ^f^Group BMSCs vs. PBS.

Correlation coefficients between MRI and histological/functional indexes of the distal stumps of injured nerves were shown in Table 6. Among several noninvasive MR parameters, FA (*r* = 0.901–0.989, *P* < 0.05) and RD (*r* = −0.928– −0.983, *P* < 0.05) had higher correlation coefficients with pathological and functional findings than the other MRI indexes.

**Table 6:** Correlation coefficient between MRI and histological indexes of the distal stumps of injured nerves.

	**FA**	**RD**	**AD**	**ADC**	**T2**
POAA	0.974	−0.973	0.913	−0.612	−0.901
	(0.000)	(0.000)	(0.000)	(0.004)	(0.000)
POMA	0.970	−0.983	0.929	−0.492	−0.920
	(0.000)	(0.000)	(0.000)	(0.027)	(0.000)
POMDA	−0.989	0.978	−0.872	0.912	0.985
	(0.000)	(0.000)	(0.000)	(0.000)	(0.000)
TOM	0.901	−0.928	0.908	−0.430	−0.750
	(0.000)	(0.000)	(0.000)	(0.059)	(0.000)
DOMF	0.921	−0.945	0.911	−0.463	−0.797
	(0.000)	(0.000)	(0.000)	(0.040)	(0.000)
SFI	0.960	−0.968	0.868	−0.838	−0.955
	(0.000)	(0.000)	(0.000)	(0.000)	(0.000)

In the four subgroups, there is a good correlation between the T2, FA, fractional anisotropy; RD, radial diffusivity; AD, axial diffusion; ADC, apparent diffusion coefficient; POAA, values of the distal segments of injured nerves with the percentage of axonal area; POMA, percentage of myelin area; POMDA, percentage of myelin debris area; TOM, thickness of myelin; DOMF, diameter of myelinated fibers; and SFI, sciatic functional index.

## Discussion

In a rat model of sciatic nerve crush damage, we found that combined BMSCs and EA therapy synergistically improved axon and myelin regeneration. Moreover, the combined therapy significantly decreased post-injury nerve edema, improved the axon guiding factor, and expedited motor function recovery. These longitudinal alterations can be evaluated quantitatively and morphologically *in vivo* by MRI. These findings expand our understanding of the neurogenic mechanisms mediated by EA-BMSCs as a potential treatment target for PNI and provide vital information for treating this disease in humans in the future.

BMSCs may exert therapeutic effects in experimental PNI models by multiple pathways, including stimulation of angiogenesis and neurogenesis, generation of neuroprotective and neurotrophic chemicals, differentiation into repair-relevant cell types, and modulation of the immune system (Moattari et al., 2018; Lavorato et al., 2021). According to our data, however, even untreated injury-model rats (PBS group) displayed nerve self-healing potential. The BMSCs alone treatment group did not demonstrate a substantial functional recovery advantage. Similar results were obtained in our previous study, showing that the BMSCs group promoted regeneration only at the early stage and then exhibited a similar trend to the control group (Chen et al., 2017).

Several factors may contribute to the inadequate efficacy of stem cell therapy in PNI. A small percentage of the transplanted BMSCs survived for up to 8 weeks. Several of the surviving cells developed into Schwann cells. BMSCs might exert their therapeutic effect *via* paracrine factors rather than *in vivo* transdifferentiation into the target cell (Duan et al., 2017). Furthermore, BMSCs could elicit a humoral and cellular immune response *in vivo* and promote inflammation when the immune system is under-activated (Ankrum et al., 2014; Jiang and Xu, 2020). This might explain the slower rate of edema reduction and debris removal in the stem cell-alone group.

In our investigation, combining BMSCs with EA yielded the most effective therapeutic outcomes, including optimal myelin and axonal regeneration and recovery of nerve function. The combination suggests that EA not only increases the regeneration of myelin and axons on its own but also enhances the efficacy of stem cells by improving their survival, migration, paracrine function, and neural differentiation. In terms of several histological parameters of nerve fiber regeneration and the sciatic nerve function index, the EA-only group demonstrated superior or equal efficacy compared to the stem cell-only group. It also demonstrates that electroacupuncture has distinct benefits in treating peripheral neuropathy, is non-invasive and inexpensive, and should be promoted as a therapy approach.

Netrin-1 has a significant chemotropic role in axon guidance, cell migration, morphogenesis, and angiogenesis. It was the first axon guidance molecule found in vertebrates. Schwann cells, the cell bodies of sensory neurons, and the axons of both motor and sensory neurons express netrin-1 receptors in the peripheral nervous system. Schwann cells direct regenerated axons to their targets by nerve gap migration and the formation of nerve bridges. Without Schwann cell guidance, regenerated axons lack directionality and travel along ectopic paths following peripheral nerve transection injury (Dun and Parkinson, 2017). Given the evidence from many studies showing that netrin-1 is associated with Schwann cells and undergoes a regeneration-related increase, netrin-1’s influence on peripheral nerve regeneration will be complex.

Netrin-1 protein is expressed in the myelin sheath composed of Schwann cells, therefore when it is secreted, the corresponding myelin is colored and outlined. In the present study, the netrin-1 protein expression was upregulated 1 and 2 weeks after rat sciatic nerve injury, followed by a rapid decrease at 3 weeks, then a gradual decrease to a normal level, consistent with available reports (Lee et al., 2007). The substantial rise in netrin-1 expression might be connected with the proliferation of Schwann cells. These cells are the primary source of netrin-1 protein in the peripheral nerve (Madison et al., 2000; Ellezam et al., 2001; Webber et al., 2011). Notably, netrin-1 can promote Schwann cell proliferation and migration (Lee et al., 2007; Lv et al., 2015). As the degree of sciatic nerve injury increases, netrin-1 secretion also does. In a study comparing a crush injury and a transection injury model, netrin-1 protein could not become detectable 2 weeks after a crush injury, inconsistent with our results. The reason was that our crush injury model had a more severe degree of damage (Madison et al., 2000). Additionally, we found that netrin-1 in the EA + BMSCs and EA groups was higher than in the BMSCs and control (PBS) groups at 1–3 weeks; all groups tended to be at a normal level at 4 and 6 weeks.

The results suggested that EA treatment could promote netrin-1 secretion, which decreased with recovery from the injury. It has been reported that EA promotes nerve regeneration and functional recovery. The mechanism might be associated with the enhancement of Schwann cell proliferation and the upregulation of nerve growth factors (Hu et al., 2018). Moreover, our data showed that at 1–3 weeks, the expression of netrin-1 was significantly increased in combined BMSCs and EA treatment compared to controls or BMSCs alone, and this was likely because EA treatment enhances BMSCs to secrete netrin-1, thereby improving the viability of BMSCs (Ke et al., 2015). The above results indicated that EA and BMSCs have a synergistic effect.

The amount of *in situ* axonal regeneration was evaluated using multiparametric MRI. T2 FA and RD values can serve as sensitive indicators for detecting peripheral nerve injury and non-invasive instruments for monitoring nerve regeneration. T2 hyperintensity of the distal degenerating nerves is caused by an increase in water content, myelin turnover, vascular permeability, inflammatory mediators, and axonal and myelin degradation products (Shen et al., 2010; Chen et al., 2015). In this study, the rapid recovery of T2 values and nerve diameter in injured nerves treated with EA at 2–3 weeks indicated that fewer nerves developed post-injury inflammatory edema during the early stages of nerve injury, which might be associated with the treatment of EA. Simultaneously, histological assessments showed that a higher debris removal rate was evident within the EA and EA + BMSCs subgroups. The result indicated that EA and stem cell therapy were anti-inflammatory and optimized the regenerative environment. More importantly, EA may have reduced graft rejection after stem cell transplantation while enhancing the function of stem cells in immune regulation.

Quantitative DTI metrics such as FA and RD values can be further compared with histological results and behavioral assessments for a fuller picture of nerve regeneration (Chen et al., 2017). Compared to BMSCs alone and the control group (PBS), the FA and RD values of injured nerves treated with EA and EA + BMSCs indicated rapid nerve tissue repair in this study. As histological evaluations revealed thicker myelin, thicker axons, and a higher concentration of the axon guidance molecule (netrin-1) in regenerating nerve fibers of the EA + BMSCs group compared to the EA or BMSCs-alone group, the rapid recovery of FA and RD values were likely attributable to enhanced axonal regeneration. Furthermore, FA values, which are the most important quantitative parameters of DTI, correlate more strongly with axon packing density and diameter than with axon diameter alone (Takagi et al., 2009).

In addition, FA assesses the degree of anisotropic diffusion, which represents the degree of cell alignment in nerve fiber pathways (Chen et al., 2017). In our study, the FA value began to grow 2 weeks after sciatic nerve injury, with the most significant increase occurring between weeks 2 and 4 in the EA + BMSCs group. The EA + BMSCs group exhibited denser and more uniformly distributed new myelin sheaths than the other groups, with the EA group next. RD values show myelin integrity and correlate most strongly with myelin thickness and axon regeneration (Martín et al., 2017). At week 2, we found that RD recovered faster than FA, probably because of the more sensitive RD value, on the one hand, reflecting the efficacy of electroacupuncture combined with stem cells in promoting myelin regeneration at the early stage of nerve regeneration on the other (Chen et al., 2017, 2022).

Axial diffusivity (AD) characterizes the mean diffusion coefficient of water molecules diffusing parallel to the tract in the voxel of interest (Song et al., 2003). We discovered that AD was not the same at week 6 as other MRI parameters, which did not return to normal at week 6, in contrast to pathological indicators and SFI. Although toluidine blue staining at week 6 suggested that fiber diameter and myelin thickness were substantially restored, electron microscopic ultrastructure revealed that the PBS group had more aberrantly regenerated myelinated fibers, displaying wrinkling, and central axon occlusion, which may have contributed to the failure of AD to recover (Mirzakhani et al., 2018; Liu et al., 2019). This was consistent with the trend of the percentage of axon area, further demonstrating that AD was closely related to axons and AD can respond to axonal regeneration.

Among these DTI metrics, FA and RD had higher correlation coefficients with pathological and functional findings than the others. Thus FA and RD might be more sensitive and stable biomarkers to evaluate nerve repair.

Our study had several limitations. First, SFI primarily reflected the recovery of motor function, whereas neurosensory recovery could not be described in animals, and thermal withdrawal latency should be added in the future. Second, due to the very small specimen volume of the injured nerve and low expression of netrin-1 protein, the protein quantitative assay such as Western blot was difficult to succeed. Third, due to the high cost of TEM photographs, we were unable to analyze a large number of TEM images in this study. Future research will be required to determine the significance of changes in TEM ultrastructure for the interpretation of MRI parameter changes.

To summarize, our findings indicated that BMSCs combined with EA treatment provided a good treatment option for peripheral nerve repair. EA treatment promotes the orderly regeneration of nerves and enhances the therapeutic effect of stem cells. Multiparametric MRI, including T2-mapping and DTI, was used to monitor the extent of nerve regeneration dynamically and evaluate *in vivo* different therapeutic effects.

## Data Availability Statement

The original contributions presented in the study are included in the article, further inquiries can be directed to the corresponding author.

## Ethics Statement

The animal study was reviewed and approved by Animal Care and Use Committee of Jennio Biotech Co., Ltd.

## Author Contributions

YC: data collection and analysis, experiment design, and writing of the manuscript. ZP: statistical analysis of data and creation of statistical charts. FM: rat MRI scan, sequence optimization, and technical assistance. QX: pathological staining and analysis. LH: image processing. QL: MRI data acquisition. XY: pathological staining. YW: discussion and revision of the manuscript. XL: full access to all the data in the study and responsible for the integrity of the data and the accuracy of the data analysis. All authors contributed to the article and approved the submitted version.

## Abbreviations

AD: axial diffusion
ADC: apparent diffusion coefficient
BMSC: bone mesenchymal stem cells
DOMF: diameter of myelinated fiber
DTI: diffusion tensor imaging
EA: electroacupuncture
EIT: experimental intermediary toe spread
EPL: experimental print length
ETS: experimental toe spread
FA: fractional anisotropy
IOD: integrated optical density
MRI: magnetic resonance imaging
NIT: normal intermediary toe spread
NPL: normal print length
NTS: normal toe spread
PBS: phosphate-buffered saline
PNI: peripheral nerve injury
POAA: percentage of axon area
POMA: percentage of myelin area
POMDA: percentage of myelin debris area
RD: radial diffusivity
ROI: region of interest
SD: standard deviation
SFI: sciatic functional index
TOM: thickness of myelin.
